# Concordance between Different Criteria for Self-Reported Physical Activity Levels and Risk Factors in People with High Blood Pressure in a Rural District in Bangladesh

**DOI:** 10.3390/ijerph181910487

**Published:** 2021-10-06

**Authors:** Fakir M. Amirul Islam, Jahar Bhowmik, Donny M. Camera, Ralph Maddison, Gavin W. Lambert

**Affiliations:** 1 School of Health Sciences, Swinburne University of Technology, Melbourne, VIC 3122, Australia; jbhowmik@swin.edu.au (J.B.); dcamera@swin.edu.au (D.M.C.); glambert@swin.edu.au (G.W.L.); 2 Organization for Rural Community Development (ORCD), Dariapur, Narail 7500, Bangladesh; 3 Institute for Physical Activity and Nutrition (IPAN), Faculty of Health, Deakin University, Geelong, VIC 3220, Australia; ralph.maddison@deakin.edu.au; 4 Iverson Health Innovation Research Institute, Swinburne University of Technology, Melbourne, VIC 3122, Australia

**Keywords:** rural area, Bangladesh, self-reported physical activity, difference, sensitivity and specificity

## Abstract

Self-reported assessment of physical activity (PA) is commonly used in public health research. The present study investigated the concordance of self-reported PA assessed using the global physical activity questionnaire (GPAQ) and two different measurement approaches. Participants (*n* = 307, aged 30–75 years with hypertension) were recruited from a rural area in Bangladesh. We analyzed the difference between the World Health Organization (WHO) recommendations of more than 600 metabolic-equivalent time-minutes (MET-min) and the self-reported active hours, at least 2.5 h per week. Tests of sensitivity and specificity were conducted to determine concordance between the two measures. According to the WHO criteria, 255 (83%) participants were active more than 600 MET-min per week and 172 (56%) people were physically active 2.5 h or more per week, indicating a 27% difference in self-reported PA. The sensitivity, specificity, positive and negative predictive values and concordance between the two measures were 64%, 92%, 98%, 34% and 70%, respectively. Considering the WHO MET-min as the appropriate measure, 89 (35%) were false negative (FN). Older age, professionals and businesspersons were associated with a higher proportion of FN. There is a gap between self-reported PA, thus a better estimate of PA may result from combining two criteria to measure PA levels.

## 1. Introduction

Physical inactivity and sedentary behavior are important modifiable risk factors associated with hypertension and all-cause mortality [1,2,3,4,5]. Worldwide, it is estimated that physical inactivity caused 6% (ranging from 3.2% in southeast Asia to 7·8% in the eastern Mediterranean region) of the burden of disease from coronary heart disease, 7% of type 2 diabetes, 10% of breast cancer and 10% of colon cancer. Inactivity causes 9% of premature mortality or more than 5.3 million of the 57 million deaths that occurred worldwide in 2008. If inactivity was not eliminated but instead decreased by 10% or 25%, more than 533,000 and more than 1.3 million deaths, respectively, could be averted every year. The estimated increase in the life expectancy of the world’s population would be 0.68 (range 0.41–0.95) years by elimination of physical inactivity [6,7]. Estimates of physical activity often rely on self-reported responses [8,9,10,11,12], which are prone to subjective biases and are less accurate than objective measures [13]. An accurate measurement of physical activity is important in order to understand physical activity-related diseases [14] and to determine the dose–response relationship between the volume, duration, intensity and pattern of physical activity and its associated effect on health. Different approaches to measuring physical activity limit the comparability among studies and across diverse populations [15]. Individuals often over- or underestimate their physical activity levels and sedentary time [16,17,18,19,20,21]. Those who overestimate are more likely to believe that physical activity is less beneficial and express less intention to increase physical activity than those who are realistic about their activity [16,22].

In low- and middle income countries (LMICs), the prevalence of self-reported physical activity has been reported to be high, 86% in India [9], 97% in Nepal [10], 94% in Uganda [11] and 90% in Mozambique [23]. In Bangladesh, physical activity levels have ranged from 58% to 83%, and these vary due to different factors including age, sex, education levels and occupations [8,24,25,26]. Since physical activity plays a vital role in preventing chronic lifestyle-related diseases, including hypertension, which is common among Bangladeshi people [27,28], a reliable instrument for assessing physical activity is required. The Global Physical Activity Questionnaire version 2 (GPAQ-2) [29] was endorsed by the World Health Organization (WHO) for its STEP-wise Approach to Chronic Disease Risk Factor Surveillance (STEPS) with special consideration of key physical activity domains in developing countries [30]. Its reliability and validity has been widely studied in developing countries [31,32]. The GPAQ-2 questionnaire [33,34] is used for the collection of self-reported physical activity and requires recall of activity from the previous week. Previous studies have typically obtained self-reported physical activity by a single approach, reporting mainly as MET-min per week [8,24,26]. Islam [8] estimated metabolic equivalent task-minute (MET-min) per week and reported that 83% of people with hypertension in a rural district in Bangladesh were physically active. However, when physical activity was assessed by direct questioning about individuals’ weekly active hours, this percentage fell to 56%. Self-reported physical activity needs to be validated with reliable and objective measures, but objective measures using accelerometers and other expensive devices are resource-intensive, limiting their use in studies in LMICs. Therefore, obtaining a better estimate of self-reported physical activity using a single questionnaire is important, especially in resource-poor settings. The current research aimed to assess (i) the difference between MET-min per week and self-reported active hours more than 2.5 h per week using the GPAQ-2 questionnaire [34] and (ii) to report the sensitivity, specificity, positive and negative predictive values and concordance between physical activity measured by two self-reported measures among adults with hypertension in a rural area in Bangladesh.

## 2. Materials and Methods

### 2.1. Study Location

The study involved baseline data collected as part of a cluster randomized controlled trial (RCT) conducted with 307 participants aged 30–75 years in the Banshgram Union of the Narail District in Bangladesh. The trial registration is ClinicalTrials.gov, NCT04505150, registered on 7 August 2020. Participants from the cross-sectional Bangladesh Population-based Diabetes and Eye Study [35,36], who were previously diagnosed with stage 1 hypertension [37], were the source for the current investigation. The study area was divided into two clusters consisting of 154 participants from cluster 1 and the remaining 153 participants from cluster 2. Cluster 1 was the intervention cluster and cluster 2 was the control cluster. The study area, the administrative units of Bangladesh and all other relevant information can be obtained elsewhere [38,39].

### 2.2. Statistical Power

The sample size 307 was adequate to estimate a difference of 27% with a 5% margin of error with at least 90% power at a two-sided significance level of 0.05 [40].

### 2.3. Recruitment

We recruited participants from December 2020 to January 2021. Since data were collected during the COVID-19 pandemic, appropriate precautions were maintained, including social distancing and wearing facemasks. The Organization for Rural Community Development (ORCD), a local NGO in Bangladesh (www.orcdbd.org), and its investigators were involved in recruitment and data collection. The ORCD investigators and trained data collectors communicated with the potential participants over the telephone or in direct contact. Potential participants were assessed for inclusion and exclusion.

The inclusion criteria were: clinic blood pressure more than or equal to 130/80 mm Hg who were not taking medication, blood pressure < 130/80 but using anti-hypertensive medication for a minimum of six weeks and permanently living in the Banshgram Union only. The exclusion criteria were: aged >75 years of age, pregnant women, advanced CVDs or had any serious condition that restricted their participation in the study. Data were collected from face-to-face interviews with the ORCD investigators and four trained data collectors who took part in data collection. An equal proportion of men and women subjects were recruited. Details of data collection and source population have been described elsewhere [36,38].

### 2.4. Ethics Approval and Consent Processes

We conducted the study following the Declaration of Helsinki. The study protocol was approved by the Swinburne University of Technology Human Research Ethics Committee (Review reference: 20202723-5020). Written informed consent was obtained from participants before data collection. The participants were assured that their participation or decision not to participate in the study would not influence their medical care or relationship with the local NGO.

### 2.5. Participant Benefits

Participation in this study was voluntary and there was no individual financial reimbursement. However, the study investigators provided 20 Omron blood pressure measuring units to the team leaders, a total of 20 teams. One machine was given per leader who monitored the blood pressure of 15 participants’ during the intervention [38]. The blood pressure devices were not taken back after the intervention, allowing future blood pressure monitoring in the community.

### 2.6. Outcome Measures

#### 2.6.1. The Difference in Self-Reported Physical Activity

We used the GPAQ-2 [34], developed by the WHO for physical activity surveillance in developing countries to measure physical activity levels. The GPAQ-2 questionnaire includes 16 questions covering moderate- and vigorous-intensity physical activity and sedentary time. Physical activity was measured based on physical activity at work, transportation (travel to and from places) and recreational activities such as participating in any sports programs. Vigorous-intensity activities were defined as “activities that require hard physical effort and cause large increases in breathing or heart rate” and moderate-intensity activities were defined as “activities that require moderate physical effort and cause small increases in breathing or heart rate”. The participants were asked whether they were involved in vigorous or moderate-intensity activities for at least 10 min continuously, if so, for how many days (frequency) they performed these activities in a typical or usual week and for how much time (intensity) they spent on a specific day [34]. People who spent less than 2.5 h of physical activity per week were considered to have low physical activity levels. The total physical activity, comprised of work, commuting and recreation activities, was converted to metabolic equivalent task (MET) minutes per week, weighted by GPAQ-assigned MET energy expenditure ratios per kilogram per hour of 4 for moderate and 8 for vigorous-intensity activities. MET is the unit used to express the intensity of physical activities. The methods for MET calculation have been described elsewhere [26,41]. The GPAQ analysis guide has explained the MET-min calculation in detail [34]. The difference in reporting physical activity was measured from the participants who reported as being active based on MET-min per week and at least active for 2.5 h per week using the same GPAQ-2 questionnaire [34].

#### 2.6.2. Concordance in Physical Activity Measure

Concordance is the overall probability that the physical activity of a person is correctly classified. First, we computed the sensitivity, specificity, positive and negative predictive values [42]. Sensitivity: the probability that people acknowledged that they were at least 2.5 h active “a” given that they followed the WHO recommenced guidelines to be active “a + b”, i.e., a/(a + b). Here, “a” is the true positive and “b” is a false negative.

Specificity: the probability that people acknowledged that they were less than 2.5 h active “d” given that they were unable to follow the WHO recommenced guidelines to be active “c + d”, i.e., d/(c + d). Here, “d” is the true negative and “c” is a false positive.

Positive predictive value (PPV): the probability that people followed the WHO recommended guidelines given that they acknowledged that they had at least 2.5 h of activity per week and was calculated using the formula:

$$\mathrm{PPV}=\frac{Sn\times P}{Sn\times P+\left(1-Sp\right)\times \left(1-P\right)}$$

Negative predictive value (NPV): the probability that people did not follow the WHO recommended guidelines given that they acknowledged that they had less than 2.5 h of activity per week and was calculated using the formula:

$$\mathrm{NPV}=\frac{Sp\times \left(1-P\right)}{\left(1-Sn\right)\times P+Sp\left(1-P\right)}$$

Concordance was defined as the overall probability that a person correctly classified their physical activity levels:

Concordance = *Sn* × *P* + *Sp* × (1 − *P*)

where *Sn* = Sensitivity, *Sp* =Specificity, *P* = prevalence.

### 2.7. Sociodemographic Factors

Sociodemographic factors included age, gender, the highest level of education (categorized as no schooling, primary to high school (grade 1 to 9), secondary school certificate or any higher-level education) and socioeconomic status (classified as poor and middle class or rich), assessed according to Cheng et al. [43]. The current study categorized occupations as farmer, homemaker, self-managed business, laborers that include digging soils, pulling a rickshaw or any laborious works, and government and non-government employees.

### 2.8. Questionnaire Rigor and Preparation

We first prepared the questionnaire in English and then translated it into Bengali by a local senior educator and the principal investigator. The two translated versions were then combined and finalized with the questionnaire with an agreement of two translators to use for data collection. The questionnaire was tested with ten adults with hypertension who were not included in the final study. Questionnaire rigor and preparation have been described elsewhere [38].

### 2.9. Statistical Analysis

Proportions of participants taking at least 2.5 h of physical activity per week and those less than 2.5 h per week were reported in relation to participants’ characteristics, including sex, age, level of education and occupation, using bivariate analysis (Chi-square tests). Sensitivity was calculated from those who were reported to be at least 2.5 h active, given that they were active according to WHO recommended guidelines [44]. Specificity was calculated from those who were active less than 2.5 h, given that they also did not follow the WHO recommended guidelines [44]. The positive predictive value was defined based on those who fulfilled the WHO recommended guidelines, given that they were at least 2.5 h active. The negative predictive value was calculated from those who did not follow the WHO recommended guidelines to be active, given that they were less than 2.5 h active. Binary logistic regression analysis was performed to report the odds ratio (95% confidence interval) for the outcome variable of false negative physical activity among the participants who were reported physically active according to the WHO recommended guidelines. Statistical software SPSS (IBM SPSS Statistics for Windows, Version 27.0. Armonk, NY: IBM Corp) was used for statistical analyses. MedCal [45] was used to compute the sensitivity, specificity, positive and negative predictive values and the concordance of self-reported physical activity.

## 3. Results

Overall, according to WHO recommended MET-min ≥600 per week, 83% of participants were physically active. In response to sedentary behavior in the same GPAQ questionnaire, 56% of participants reported that they were physically active for more than 2.5 h per week. Therefore, the difference between these two approaches in measuring physical activity was 27%. Although the difference tended to be higher in men, the older age group, employees and businesspersons, none of these differences were significant (Table 1).

**Figure 1:**
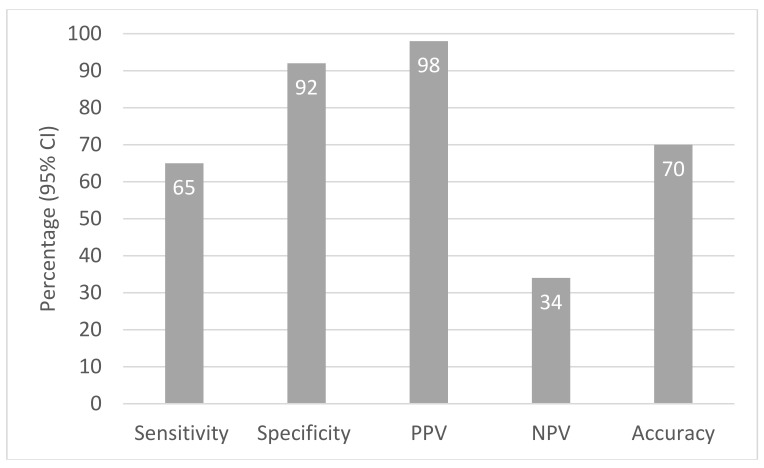
Sensitivity, specificity, positive predictive value (PPV), negative predictive value (NPV) and concordance in the total sample.

Considering that the prevalence of physically active individuals was 83% according to the MET-min per week criteria, 35% (89/257) were false negative and 2% (4/172) were false positive. The sensitivity (95% confidence interval (CI)) of the MET-min per week was 65 (59, 71)%, specificity (95% CI) 92 (81, 98)%, positive predictive value (95% CI) 98 (94, 99)%, negative predictive value (95% CI) 34 (30, 38)% and concordance (95% CI) was 70 (64, 75)% (Figure 1). Sensitivity, specificity and positive predictive value were higher for people who had high physical activity levels ≥3000 MET-min per week compared to those who had physical activity levels 600–2999 MET-min per week. The concordance (95% CI) of physical activity levels was 77% (71, 82) for people with ≥3000 MET-min per week compared to 45% (36, 55) for people with 600–2999 MET-min per week (Table 2).

WHO recommended physical activity (83%) is considered to be the prevalence of physical activity and self-reported at 2.5 h physical activity is used as a surveillance tool. Physical activity MET-min <600 equals 50 was used for both moderate and high physical activity. Total N = 108 + 249 − 50 = 307. PPV was calculated using the formula in the methods section. For sensitivity 0.36 and prevalence 0.83, the PPV = (0.36 × 0.83)/((0.36 × 0.83) + (1 − 0.92) × (1 − 0.83)) = 0.96. Similarly, for specificity 0.92 and prevalence 0.83, the NPV = (0.92 × 0.17)/((0.92 × 0.17) + (1 − 0.36) × 0.83)) = 0.22.

Table 3 presents factors associated with the reported false negative physical activity according to WHO recommended MET-min >600 per week. Older adults aged 60–69 years (odds ratio (OR) = 3.30; 95% CI = 1.40, 7.74) and 70–75 years (OR = 5.73; 95% CI = 1.58, 20.7) were associated with a higher proportion of false negative physical activity than younger adults of age 30–40 years. After adjustment for age and education levels, homemakers (OR = 3.06, 95% CI = 1.40, 6.69), professionals (OR = 8.02, 95% CI = 2.92, 22.2) and businesspersons (OR = 5.46, 95% CI = 1.81, 16.5) were associated with a higher proportion of false negative physical activity compared to farmers. Only occupation was adjusted for age and education levels as this was the only variable that showed significant association with false negative physical activity. Sex was not included in the adjusted model as participation in some category levels were sex specific, for instance, farmer was zero for women and homemaker was zero for men. Sex, education levels, socioeconomic condition or diabetes status were not found to be associated with false negative physical activity.

## 4. Discussion

We sought to determine the concordance between two self-reported measures of physical activity. Key findings from this study were: (1) there was a significant difference between self-reported physical activity measured by the WHO recommended MET-min per week and self-reported active hours more than 2.5 h per week and (2) although self-reported physical activity was found to be above 80% measured by the WHO recommended MET-min >600 per week, the concordance between this and the estimation based on self-reported active hours of more than 2.5 h per week was less than three-quarters.

Self-reported questionnaires are the most commonly used tools to assess changes in physical activity. While they are cost-efficient and provide important contextual information about physical activity [8,9,10,11,22,23,24,25,26,34], they are sensitive to both overestimation and underestimation of true physical activity levels [21]. Because it is difficult to objectively measure physical activity levels at the population-level, self-reported measures are commonly used as a surveillance tool [8,9,10,11,12]. Self-reported measures are limited by recall bias and variation in reporting accuracy for different intensities and domains [46,47]. Measurement errors are common in self-reported measures, and it is recommended to perform a well-designed validation study as it allows for understanding and correcting measurement errors [48,49]. There is an increased interest in not only measuring the overall amount of physical activity undertaken but also understanding the breakdown of physical activity by domains (i.e., transportation, recreation, occupational, sports and household) so as to better understand any observed changes in self-reported responses and to better target responses [50,51].

Our study showed a difference between self-reported physical activity reported by the same participants from their answers to different questions of the same GPAQ-2 questionnaire [34]. Although this is not directly comparable, discrepancies between self-reported physical activity and objective measures have been reported in previous studies [17,18,19,20,52].

The GPAQ was reported to be an acceptable measure for physical activity surveillance in Bangladesh but reported to have a bias towards the overestimation of physical activity [32] which supports the finding from previous studies [19]. The measurement method may have a significant impact on the practical levels of physical activity. It can be observed that self-report measures of physical activity could be overestimated and underestimated when compared with directly measured physical activity levels [21].

Based on the sensitivity and specificity, our study found approximately two-thirds of rural adults were physically active, endorsed by asking two questions about participants’ physical activity levels using the same GPAQ questionnaire. The concordance was higher among people who had MET-min more than 3000 per week compared to people who had MET-min 600–2999 min per week. Although this percentage is similar to findings from other studies [24,26], the results are not directly comparable as the current study took into consideration measuring physical activity by two different questions from the same questionnaire—one for domain-specific MET-min per week and the other for sedentary lifestyle measured by active hours less than 2.5 h per week. The same questionnaire that eliminated the false positive and false negative physical activity levels compared to the previous studies considered a single approach of measuring physical activity. In this study, older adults were found to be prone to false negatives as compared to younger adults. The difference could be due to a range of factors such as health status, medical conditions and medications, fatigue, pain, concentration and distractibility, changes in mood, depression, anxiety and problems with memory and cognition [51,52]. Self-reported habitual physical activity measures rely significantly on the respondent’s ability to provide accurate physical activity behaviors. However, the findings warrant further investigation with larger samples.

Our study reported that professionals and businesspersons were associated with a higher proportion of false negative physical activity than farmers and daily laborers. A higher proportion of false negative physical activity among this group may be explained by them being routinely occupied in tasks that require less physical activity. Although they spent more than 600 MET-min per week, they were unaware that they spent at least 2.5 h in physical activity per week and hence were categorized into an inactive group. This could postulate that they are more likely to have a positive perception of physical activity, leading them to think of being inactive although they fulfil at least a minimum level of physical activity. The results are consistent with previous studies that reported that higher education levels and being professionals were associated with positive attitudes towards physical activity in managing chronic diseases, including hypertension [53,54]. Another possible reason could be that farmers and other occupations related to hard physical labor, especially in LMICs, are inclined towards being more physically active compared to professional occupations that tend to be more sedentary and thus less physically active [8,26,42,55,56,57].

The current study has significant implications. There are different methods available for the assessment of physical activity [58]. The GPAQ is one of the most used validated tools [46] and is widely used in LMICs, which is self-reported and less resource-intensive [21]. A reliable estimate of physical activity using the same GPAQ tool will help measure physical activity in Bangladesh, resource-poor settings and remote areas in developed countries. The study’s strengths include the face-to-face data collection methods and relative ease of use in large samples. The study also involved gender-balanced participation, where almost 50% of the participants were women. The study sample represented a good mix of older and younger adults. To our knowledge, this is the first study to evaluate the difference and concordance of two self-reported measures on physical activity in Bangladesh. However, the study has some limitations, such as data being collected from a single rural district which restricts generalization of the results at the national level. The Kappa statistic is typically used to test the agreement between two measures. However, in the present instance, the scales and sample sizes were different for the two measures and therefore the test of the agreement was not performed. Moreover, due to the cross-section study, causal inference about the association cannot be drawn from the survey outcome. Nevertheless, the risk of bias has to be considered when concluding our present findings.

## 5. Conclusions

There was a difference between self-reported physical activity levels measured by two different questions on the same activity of the same tool. None of the socio-demographic factors were associated with this difference. Sensitivity, specificity and concordance can provide a more reliable estimate of physical activity levels with the concordance of two measures. Health promotion programs, especially among people with reported false positive and false negative physical activity, need to be organized for participation in physical activity programs. The sensitivity and specificity results may guide future research in understanding why participants under or over-report physical activity, which could help develop better self-reported physical activity measures for developing countries.

## Figures and Tables

**Table 1:** Difference between WHO recommended MET-min ≥600 per week and self-reported physically active more than 2.5 h per week and associated sociodemographic factors among the people with high blood pressure.

	No of Participants	≥2.5 Hours Activities Per Week	WHO Recommended, MET-min >600 min/Week	Discrepancy	*p*-Value
Factors		*n* (%)	*n* (%)	*n* (%)	
Total	307	172 (56.0)	255 (83.0)	83 (27.0)	
Female	154	84 (54.5)	121 (78.6)	37 (24.1)	0.77
Male	153	88 (57.5)	134 (87.6)	46 (30.1)	
Less than 40	46	35 (76.1)	46 (100)	11 (23.9)	0.21
40–49	65	45 (69.2)	59 (90.8)	14 (21.6)	
50-59	95	57 (60.0)	81 (85.3)	24 (25.3)	
60–69	79	30 (38.0)	57 (72.2)	27 (34.2)	
70–75 years or older	22	5 (22.7)	14 (63.6)	9 (40.9)	
No education	99	48 (48.5)	70 (70.7)	22 (22.2)	0.39
Primary to high school	148	88 (59.5)	131 (88.5)	43 (29.0)	
SSC or above	59	36 (61.0)	54 (91.6)	18 (30.6)	
Poor	92	56 (60.9)	77 (83.7)	21 (22.8)	0.59
Middle class or rich	214	115 (53.7)	177 (82.7)	62 (29.0)	
Farmer	66	49 (74.2)	65 (90.3)	16 (16.1)	0.00
Homemakers	146	84 (57.5)	118 (80.8)	34 (23.3)	
Employees	53	15 (28.3)	38 (71.7)	23 (43.4)	
Businessperson	24	13 (54.2)	24 (100)	11 (45.8)	
No diabetes	217	118 (54.4)	171 (78.8)	53 (24.4)	0.32
Diabetes	41	20 (48.8)	36 (87.8)	16 (39.0)	
Unknown	49	34 (69.4)	48 (97.9)	14 (28.5)	

**Table 2:** Confusion matrix, sensitivity, specificity, positive predictive and negative predictive values with 95% confidence interval for moderate and high physical activity levels.

Physical Activity MET-min <600 min/Week vs. 600–2999 min/Week					
≥2.5 h of physical activity	Yes	No	Total	PPV (95% CI)	NPV (95% CI)
Yes	21 (a)	4 (c)	25	96 (90, 98)	
No	37 (b)	46 (d)	83		22 (18, 25)
Total	58	50	108		
Sensitivity (95% CI) %	36 (24, 50)				
Specificity (95% CI) %		92 (81, 98)			
Concordance					45 (36, 55)
Physical activity MET-min <600 min/week vs. ≥3000 min/week					
≥2.5 h of physical activity	Yes	No	Total	PPV (95% CI)	NPV (95% CI)
Yes	147 (a)	4 (c)	151	98 (95, 99)	
No	52 (b)	46 (d)	98		40 (34, 46)
Total	199	50	249		
Sensitivity (95% CI), %	74 (67, 80)				
Specificity (95% CI), %		92 (81, 98)			
Concordance					77 (71, 82)

**Table 3:** Association of sociodemographic factors with false negative physical activity levels of 257 physically active adults.

	WHO Recommended MET-min >600 Per Week		
Factors		*n* (%)	OR (95% CI) *
Total	257	89 (35)	
Sex			
Female	123	42 (34.1)	1.00
Male	134	47 (35.1)	1.04 (0.62, 1.74)
Age group, years			
Below 40	46	11 (23.9)	1.00
40–49	59	15 (25.4)	1.09 (0.44, 2.66)
50–59	81	25 (30.9)	1.42 (0.62, 3.24)
60–69	57	29 (50.9)	3.30 (1.40, 7.74)
70–75	14	9 (64.3)	5.73 (1.58, 20.7)
Level of education			
No education	70	23 (32.9)	1.00
Primary to high school	133	47 (35.3)	1.12 (0.61, 2.06)
SSC or above	54	19 (35.2)	1.11 (0.52, 2.35)
Socioeconomic status			
Poor	78	22 (28.2)	1.00
Middle class	178	67 (37.6)	1.54 (0.86, 2.74)
* Occupation			
Farmer	65	13 (20.0)	1.00
Homemakers	120	39 (32.5)	1.93 (0.94, 3.95)
Professionals *	38	23 (60.5)	6.13 (2.52, 14.9)
Businesspersons **	24	11 (45.8)	3.38 (1.24, 9.27)
Diabetes status			
No diabetes	173	58 (33.5)	1.0
Diabetes	36	17 (47.2)	1.77 (0.86, 3.67)
Unknown	48	14 (29.2)	0.82 (0.41, 1.64)

* OR (95% CI): 6.76 (2.38, 19.2) and ** OR (95% CI): 5.26 (1.74, 15.9) are adjusted for age, sex and level of education.

## Data Availability

Data will be made available on reasonable request from the corresponding author.
